# Gelatin Hydrogels Reinforced by Absorbable Nanoparticles and Fibrils Cured *In Situ* by Visible Light for Tissue Adhesive Applications

**DOI:** 10.3390/polym12051113

**Published:** 2020-05-13

**Authors:** Shih-Min Wei, Ming-Ying Pei, Whei-Lin Pan, Helmut Thissen, Shiao-Wen Tsai

**Affiliations:** 1 Graduate Institute of Biomedical Engineering, Chang Gung University, Taoyuan 333, Taiwan; sallykitty27@gmail.com; 2 Department of Biomedical Sciences, Chang Gung University, Taoyuan 333, Taiwan; b0309131@cgu.edu.tw; 3 Department of Periodontics, Chang Gung Memorial Hospital, Taipei 105, Taiwan; helen481209@gmail.com; 4 Commonwealth Scientific and Industrial Research Organisation (CSIRO) Manufacturing, Clayton, VIC 3168, Australia; Helmut.Thissen@csiro.au

**Keywords:** photo-crosslinking, hydrogel, reinforcement, sealant

## Abstract

Most gelatin hydrogels used in regenerative medicine applications today are fabricated by photocrosslinking due to the convenience and speed of this method. However, in most cases photoinitiators are used, which require UV light, which, in turn, can cause cell and tissue damage, or using functionalized gelatin. Recently, ruthenium (II) tris-bipyridyl chloride has been studied as an initiator that can induce dityrosine bond formation using visible light. In addition, continuous fibrils and small particles are often used to reinforce composite materials. Therefore, this study investigated the visible-light-induced photocrosslinking of native gelatin molecules via dityrosine bonds formation as well as gel reinforcement by collagen fibrils and mesoporous bioactive glass (MBG) particles. The results show that collagen and MBG exerted a synergistic effect on maintaining gel integrity with a dental LED curing light when the irradiation time was shortened to 30 s. Without the two reinforcing components, the gel could not form a geometric shape stable gel even when the exposure time was 120 s. The shear strength increased by 62% with the collagen and MBG compared with the blank control. Furthermore, our results demonstrate that the addition of collagen and MBG enhanced gel stability in an artificial saliva solution. These results demonstrate the considerable advantages of using tyrosine-containing biomolecules, and using a dental LED curing light for the crosslinking of hydrogels in terms of their suitability and feasibility for use as bioadhesives in confined clinical working space, such as the oral cavity, and in application as in situ-crosslinked injectable hydrogels.

## 1. Introduction

To date, many different methods have been proposed for guiding bone regeneration, especially in the context of dental tissue regeneration, with the aim of restoring or replacing missing oral tissue. For example, deproteinized organic bovine bone mineral has been combined with membranes and the release of growth factors for this purpose [1,2]. However, a few problems are associated with these materials, including migration of the grafted materials after implantation, and the need to remove the nonabsorbable membrane. In addition, for any dental-related disease treatment, the aqueous environment found in the dental cavity must be a concern. To overcome these difficulties, methods such as suturing materials with soft tissue or using biodegradable membranes are generally used. However, for the materials to be firmly attached to the native tissue, a different approach would be advantageous, and herein we propose a new biodegradable, gelatin-based adhesive that can be cured in situ under aqueous conditions.

The roles of tissue adhesives are not limited to stopping bleeding and closing wounds. Adhesives are also often expected to act as sustained release carriers and as temporary scaffolds for tissue regeneration. Hence, the requirements for tissue adhesives include adequate viscosity to prevent drainage from the wound, easy application, rapid gel formation on demand (fast phase transformation from solution to gel), usability in an aqueous environment, and appropriate mechanical strength, biocompatibility and biodegradability [3]. Various tissue glues are widely used in the clinic, such as fibrin glue, cyanoacrylate-based adhesives, protein-based adhesives, polyethylene glycol (PEG)-based adhesives and polyurethane-based adhesives [4,5,6,7]. Every type of glue has its unique advantages and disadvantages; for example, fibrin glue provides excellent biocompatibility but poor mechanical strength and a high risk for infection [8]. PEG-based adhesives also have poor mechanical properties; thus, they are generally used as an adjuvant glue for sutures [9,10]. In contrast, cyanoacrylate-based adhesives have poor biocompatibility and high adhesion strength and stiffness, which restrict their use in elastic soft tissues due to a mismatch in mechanical properties [6,11]. TissueGlu^®^ is a polyurethane-based adhesive, where the prepolymer solution can react with amine groups from the tissue or with water in the wound to achieve wound closure [12]. Protein-based adhesives utilize chemical-reagent-mediated crosslinking between the protein and tissues. However, to date, there has not been a universal adhesive because of the various environments in which adhesives are applied.

The use of crosslinked biopolymers for biomedical and tissue glue purposes has increased substantially in recent decades. One of the most commonly used biopolymers is gelatin. As gelatin is derived from collagen, it has separate single peptide chains; therefore, gelatin has well-known biocompatibility and biodegradability. Because of its high water absorption and viscoelasticity, gelatin is used in a wide range of medical, pharmaceutical and biotechnological applications. The crosslinking of gelatin is one of the methods used to fabricate hydrogels with high durability [13,14]. The mechanical strength of crosslinked gelatin-based hydrogels depends on the gelatin and crosslinking reagent concentrations and the degree of crosslinking [15,16,17]. Crosslinking gelatin in conjunction with other biomolecules, such as fibrinogen and chitosan, for use as adhesives or hydrogels has been reported in numerous studies [18,19,20,21,22]. To our knowledge, most studies on the in situ crosslinking of gelatin with light in the visible range have used chemically modified gelatin, such as gelatin methacrylate [5,23,24,25,26,27], rather than native gelatin molecules. In addition, gelatin hydrogels often have insufficient mechanical strength, and the chemical modification may have an intrinsic cytotoxicity. In other words, a balance between the amounts of chemical reagents used and the biocompatibility of gelatin must be maintained between reducing the crosslinking reagent content and reducing the mechanical strength. Thus, in the present work, we utilized the reinforced matrix strength concept to improve mechanical strength without increasing cytotoxicity.

Collagen is a major structural protein in mammalian animals and consists of fibrils with a semicrystalline structure. In the extracellular matrix (ECM), various proteoglycan molecules form a hydrated gel surrounding the collagen fibrils, resisting stress and preventing tissues from pulling apart. This structure indicates that the collagen fibrils play a reinforcing role in the ECM. In addition, continuous fibers are often used to reinforce the strength of composite materials because the applied load can transfer from the weaker matrix to the stiffer fibrils [28,29]. In composite metal matrices, particulates materials are commonly used to reinforce materials because they can prevent dislocation movement or serve as heterogeneous nucleation sites for precipitate formation. The extra particulates are typically much stiffer than the matrix, and therefore a significant fraction of the stiffness of the composite can be due to the reinforcing particulates. A few studies have been utilized the concept to reinforcing hydrogel [30,31,32,33]. Mesoporous bioactive glass (MBG) is a silica-based synthetic material with bioactivity and degradability. Mesoporous structures have achieved widespread use as drug carriers in sustained release applications. Compared with that of nondegrable bioceramics, such as hydroxyapatite and beta-tricalcium phosphate, the release of Ca, P, and Si ions from MBG has been demonstrated to stimulate osteoblast cell proliferation and differentiation [34,35].

Several methods have been published on the production of crosslinked gels in aqueous solutions, such as by mediation with chemical reagents (glutaraldehyde and 1-ethyl-3-(3-dimethylaminopropyl)carbodiimide, EDC) [36,37], gamma radiation [38,39,40], enzymatic reactions [41,42] and photopolymerization [43,44,45]. Molecules that contain double bonds are essential for polymerization via free radical reactions, which are generally initiated by heat [46,47], irradiation (UV or visible light) [39,48], or a metal-containing catalyst [49,50]. In some disease treatments, such as those in the dentistry, the space is limited, and in combination with the fact that high temperatures are harmful to the tissues. Thus, light irradiation is considered a superior method to heat to initiate free radical polymerization reactions if the wavelength is located within the visible range. Elvin et al [51] reported that ruthenium(II) trisbipyridyl chloride (Ru(II)bpy_3_^2+^) exposed to light can be oxidized to Ru(III)bpy_3_^3+^ in a persulfate anion-containing aqueous solution. Then, Ru(III)bpy_3_^3+^ can react with tyrosine from the protein to form a crosslinked dityrosine bond [52,53]. Following this mechanism, materials derived from recombinant collagen with high elasticity and high fatigue resistance were fabricated. The researchers used gelatin [54] and fibrinogen [55] crosslinked in this way to develop an elastic tissue sealant. Their report demonstrated the dityrosine presence in the crosslinked gelatin by amino acid and HPLC analysis. Thus, in this study, we used fibrils and particles, i.e., collagen and MBG, to reinforce a gelatin adhesive and investigated the influence of this reinforcement on the physical adhesive properties of a gelatin-based adhesive cured in situ by visible light in an artificial saliva solution, as well as the cytotoxicity and tensile strength of the gelatin/collagen composite. In clinical applications, adhesives are used not only to close wounds but also to prevent postsurgical adhesion. In particular, the adhesives used in dental disease treatment are mainly applied to enamel and dentin and are not suitable for periodontal soft tissue. Thus, we further evaluated fibroblast migration to elucidate the efficiency of our composite gel in limiting fibroblast invasion.

## 2. Materials and Methods

### 2.1. Preparation of the Photocrosslinked Adhesive

The method of MBG fabrication has previously been described [56]. Briefly, Pluronic P 123 (2.0 g) in HCl (0.5 N, 0.5 mL) was mixed with tetraethoxysilane (TEOS, 3.7 g), calcium nitrate tetrahydrate (0.49 g), and triethylphosphate (TEP, 0.34 g) at 300 rpm for 3 h. Subsequently, the MBG precursor solution was poured into a dish and kept in the hood until the solvent had completely vaporized. The collected samples were calcined at 700 °C for 3 h to eliminate the organic components and obtain the MBG. Type B gelatin and type I collagen fibrils (Victory Biotech Co., Ltd., Foshan City, China) were dissolved in phosphate-buffered saline (PBS) to obtain a 100 mg/ml solution, whereas gelatin/collagen mixtures were prepared with weight percentages of 90/10 (90G10G) and 95/5 (95G5C). Furthermore, MBG was then added and mixed well with the gelatin and collagen mixture to a content ranging from 10 to 30% (*w*/*w*). [Ru(II)bpy_3_]^2+^ chloride and sodium persulfate (SPS) were then added to the mixture to a final concentration of 1 and 20 mM, respectively. The mixture was transferred to a Teflon mold and irradiated for 30, 60, 90 and 120 s at room temperature with a dental LED curing light (LiteQ LD-107, MONITEX™, Fomed Biotech Inc., New Taipei City, Taiwan) from a distance of 1 cm. All chemicals used were purchased from Sigma-Aldrich Chemical Company (St. Louis, MO, USA) and were reagent grade unless otherwise stated.

### 2.2. Gel Stability Test

To evaluate the water absorption and stability of the gel in an aqueous environment, the gel samples, including pure gelatin, 95G5C, 90G10C and 90G10C containing 20% MBG, which were each irradiated for a designated time, and immersed in a pH 7 artificial saliva solution. The artificial saliva solution was prepared by dissolving reagent-grade CaCl_2_ (1.5 mM), KCl (130 mM), K_2_HPO_4_ (0.9 mM), and NaN_3_ (1.0 mM) in a 20 mM HEPES (4-(2-hydroxyethyl)-1-piperazineethanesulfonic acid) solution [57], and the sample wet weight was measured every 24 h. 

### 2.3. Mechanical Property Test

**Figure 1:**
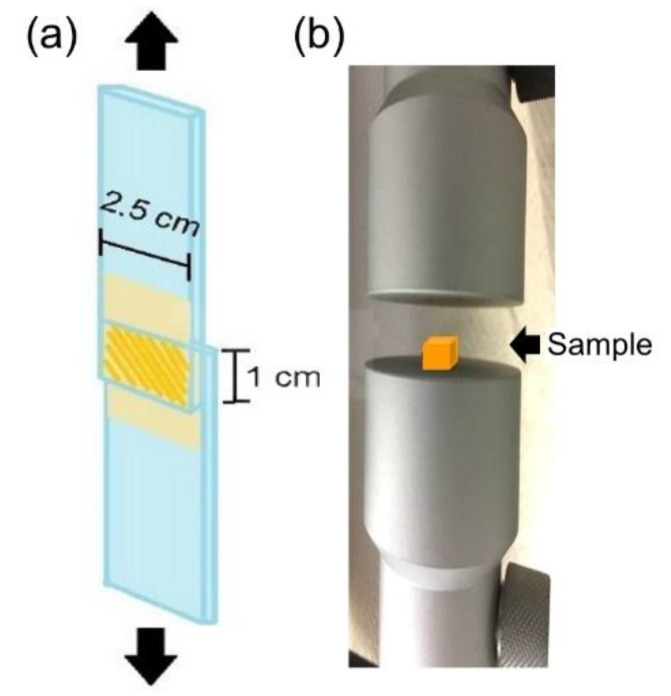
Schematically representation of the mechanical property test (**a**) shear strength test, and (**b**) compression test used in this study.

The shear strength test was performed according to reference ASTM F2255-05, a standard test method used to determine the lap-shear strength properties of tissue adhesives under tension loading by a material testing system machine (MTS, H1KT, Tinius Olsen, Horsham, PA, USA). Briefly, fresh defatted porcine skin was cut into 2.5 × 2.5 cm^2^ strips and fixed on a glass substrate with cyanoacrylate glue. We applied 100 μL of the mixed solution to the porcine skin strips, which resulted in an overlap area of 1 cm^2^. Subsequently, the overlap area was irradiated by an LED curing light at a fixed distance of 1 cm for a defined number of seconds. The two skin substrates were bonded together for an additional 10 minutes with a clamp before the two glass substrates were fixed on the specimen holder of the MTS (Figure 1a). A commercially available fibrin adhesive (EVICEL^®^, Ethicon, Inc., Somerville, NJ, USA) was used as a control. To measure the compression resistance, the gel sample was placed on the stage of the MTS (Figure 1b). The working load of the MTS machine was 50 N, and the rate was 100 mm/min. 

### 2.4. Cytotoxicity Test

The mixed solution showed a phase transformation from an aqueous solution to a gel after the photocrosslinking reaction. All of the components were entrapped within the gel. Therefore, we used an extraction method to test whether any unreacted species diffused out of the gel and affected cell viability. The cytotoxicity test was performed according to ISO 10993-5, 2009 (Biological evaluation of medical devices: Tests for in vitro cytotoxicity). In this experiment, gels were prepared with sterilized raw materials. The gel was immersed in Dulbecco’s modified Eagle’s medium supplemented with 10% fetal bovine serum (Gibco™, lot number 624294, Thermo Fisher Scientific Inc., Waltham, MA, USA) at 37 °C for 24 h. The L929 fibroblasts were cultured in 96-well tissue culture dishes (NUNC™, Thermo Fisher Scientific Inc., Waltham, MA, USA) until confluent. Then, the medium was replaced with the extract and the cells were further cultured for 24 h. Extracts from tissue culture polystyrene (TCPS) and 0.64% phenol-containing medium were used as controls. Cell viability was determined with the WST-1 reagent (Roche, Basel, Switzerland), and the resulting dye levels were measured at a wavelength of 405 nm. Images of cellular morphology were taken using an inverted microscope (Leica, Wetzlar, Germany).

### 2.5. Cell migration Measurement

An “in vitro scratch assay” was used to evaluate the capacity of the gel to prevent cell migration. Specifically, a P1000 pipet tip (Scientific Specialties, Inc., Lodi, CA, USA) was used to scrape a confluent L929 cell monolayer in a straight line to create a gap. Debris was removed by washing the cells with medium several times, and the aqueous composite was then placed within the gap and cured for 30 s. Following this, the cells were incubated at 37 °C for 24 h. Cell migration across the gap was then determined by observing the cells under a phase-contrast microscope.

### 2.6. Statistical analysis

Experiments were independently conducted in triplicate, and each time, three samples were collected in parallel. The results are expressed as the mean ± SD. Statistical analysis was performed using the SPSS v. 17 software package (SPSS Inc. SPSS Statistics for Windows, Chicago, IL, USA). All results were analyzed by the nonparametric Kruskal–Wallis H-test and, if significant at a value of *p* < 0.05, individual Mann–Whitney U-tests were conducted to determine the differences among groups. Differences of *p* < 0.05 were considered statistically significant. 

## 3. Results and Discussion

### 3.1. Characterization of the Photocrosslinking Hydrogel Reinforced by Collagen Fibrils

#### 3.1.1. Physical Properties

**Figure 2:**
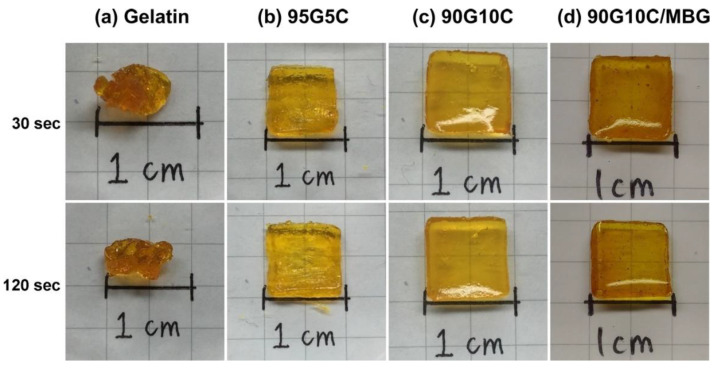
Images of various sealant compositions exposed to LED light for different durations, with (**a**) gelatin, (**b**) 95G5C, (**c**) 90G10C, and (**d**) 90G10C containing 20% mesoporous bioactive glass (MBG).

**Figure 3:**
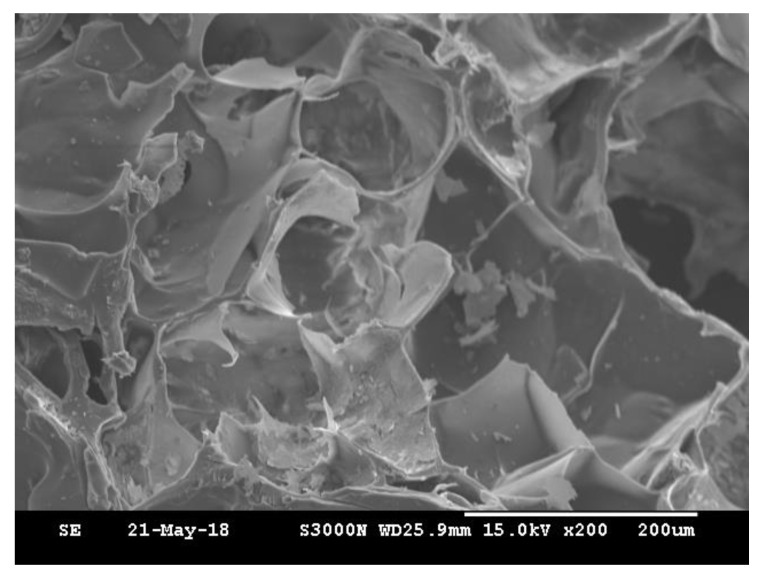
SEM micrographs of the 90G10C containing 20% MBG compositions exposed to LED light for 120 s.

The degree of photocrosslinking is known to depend on the irradiation time and the concentration of photosensitive molecules. Therefore, a shortened irradiation time will decrease the degree of photocrosslinking and may result in an incomplete sol-gel transformation. As shown in Figure 2, regardless of the irradiation time, the phase transformation from solution to gel was not complete for samples crosslinked by dityrosine formation representing gelatin only. However, the gel shape was observed to keep its integrity with increasing collagen content at the same exposure time, even for only a short (30 s) exposure time. The images indicate that, compared with gelatin alone, the additional collagen fibrils improved the geometric stability of the gel. However, the shapes of the gels with and without MBG were not significantly different (Figure 2c,d). In general, materials with higher crystallinity have higher structural stability and mechanical strength. Gelatin is denatured collagen without the typical helical structure of the protein and an amorphous structure. In contrast, collagen is a major structural protein in mammalian animals and consists of long fibrils with a semicrystalline structure [58]. Moreover, MBG is harder and tougher than gelatin and collagen. Based on these physical and structural characteristics, collagen fibrils and MBG reinforced the integrity of the gelatin matrix even after a short exposure time. The surface morphology of 90G10C containing 20% MBG hydrogel was examined via scanning electron microscope (SEM, HITACHI, S-3000N, Tokyo, Japan) at 15 kV. The image (Figure 3) indicated that MBG did not alter the formation of a high-porosity matrix.

**Figure 4:**
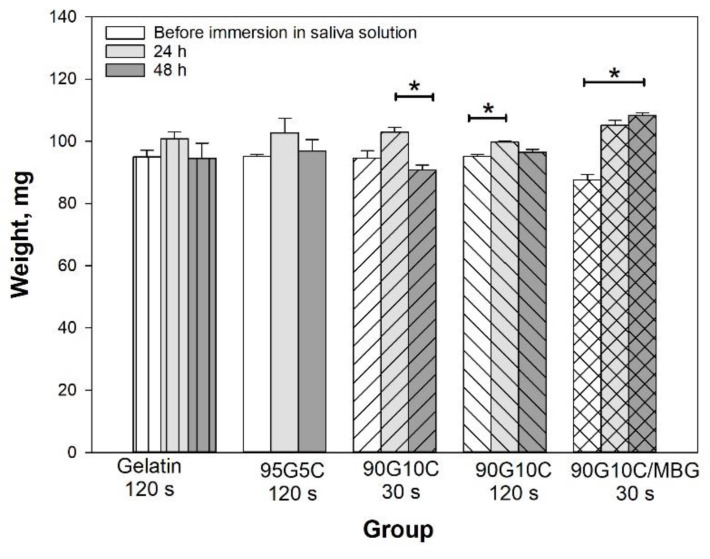
Effect of composition on hydrated gel weight. The gel samples, including pure gelatin, 95G5C, 90G10C and 90G10C containing 20% MBG, which were irradiated for a designated time, were immersed in a pH 7 artificial saliva solution, and weighted every 24 h. Data are presented as the mean ± SD (n = 9) and were analyzed using the nonparametric Kruskal–Wallis H-test. Differences of *p* < 0.05 were considered statistically significant. (*) denotes a significant difference (*p* < 0.05).

In general, the degree of hydrogel crosslinking increases with increasing irradiation time, which in turn reduces water absorption. To further investigate the effect of collagen content on gel shape stability, the gels were soaked in an artificial saliva solution for up to 48 h. The weight of all pure gelatin gels increased after being immersed in solution for 24 h, but the water absorption decreased with increasing irradiation time (Supporting Information Figure S1). However, except in the 120 s exposure group, the gel weight in all groups significantly decreased after 24 h, and the gels finally broke into small pieces if they were soaked in the solution for longer. It is difficult for a soft hydrogel to endure the hydrostatic pressure of an excessive water content due to insufficient structural strength. The data indicated that a higher degree of crosslinking limited water absorption and enhanced the maintenance of the gel shapes. However, the collagen-containing gel showed different results. Both gelatin and collagen have excellent affinity for water molecules; thus, the gel weights were similar after different light irradiation time and different compositions. As shown in Figure 4, the weight of the collagen-containing gels remained constant even after exposure for 30 s when immersed in the artificial saliva solution for up to 48 h. 

#### 3.1.2. Mechanical Properties

**Figure 5:**
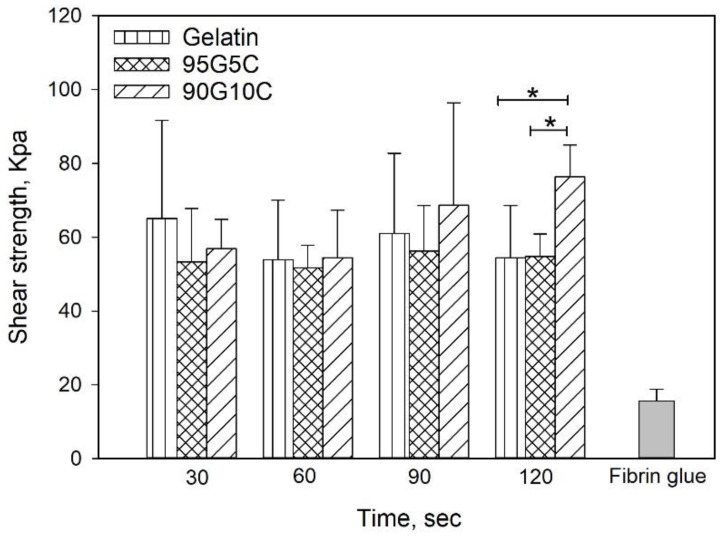
Effects of irradiation time and composition on shear strength. After 100 μL of the mixed solution was applied to the porcine skin strips, which resulted in an overlap area of 1 cm^2^. The overlap area was irradiated using an LED curing light for a designed time. Data are presented as the mean ± SD (n = 9) and were analyzed using the nonparametric Kruskal-Wallis H-test. Differences of *p* < 0.05 were considered statistically significant. (*) denotes a significant difference (*p* < 0.05).

To investigate the adhesive strength, we used fibrin glue as a control, as shown in Figure 5, and regardless of the composition and exposure time, the shear strength of all gels was significantly higher compared to the value obtained for fibrin glue. However, there was no significant difference among samples with different compositions exposed for 30, 60 and 90 s. However, the 90G10C sample irradiated for 120 s displayed a higher shear strength compared to samples with other compositions. In addition, only the shear strength of 90G10C slightly increased with exposure time. 

**Figure 6:**
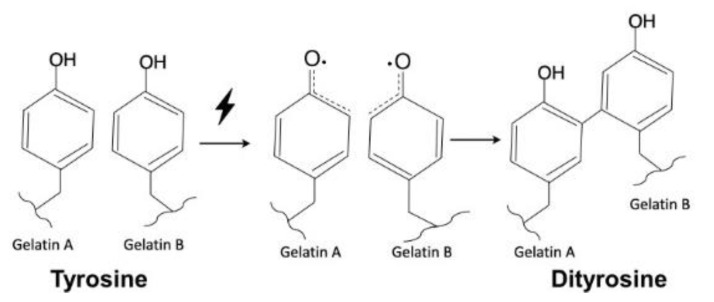
Schematic illustration of dityrosine bond formation.

The tyrosine content of 90G10C was lower than that of the other gels; therefore, a longer exposure time was needed to maintain the free radicals to achieve a reaction. The content of tyrosine in collagen is only 0.03 in 100 residues [59], and the collagen used in the study consisted of fibrils rather than triple helix molecules. In addition, a dityrosine bond can only form between two nearby tyrosine molecules. Therefore, the degree of crosslinking between gelatin and collagen or intracollagen fibrils was low (Figure 6). Although collagen fibrils cannot enhance the degree of crosslinking, their long rod-like structure reinforces the strength of the matrix in another way, improving the integrity of the matrix by 40% compared with that of gelatin. Based on the above results, 90G10C performed better than the other gels, thus, we chose 90G10C to investigate the effect of MBG for the photocrosslinkable bioadhesive application.

### 3.2. Cytotoxicity Evaluation

**Figure 7:**
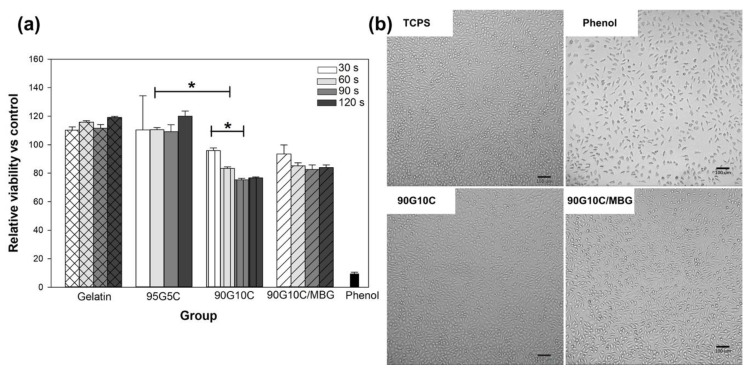
Cytotoxicity assay of various composites. The viability of L929 cells was determined with the WST-1 reagent after culture with various extracts for 24 h (**a**), and images of cellular morphology were taken using an inverted microscope (**b**). Data are presented as the mean ± SD (n = 9) and were analyzed using the nonparametric Kruskal–Wallis H-test. Differences of *p* < 0.05 were considered statistically significant. (*) denotes a significant difference (*p* < 0.05).

One of the essential requirements of materials for biomedical applications is tolerable cytotoxicity. We used methods from ISO-10993-5 to evaluate the biocompatibility of the photocrosslinked gels. Except for 90G10C, no gels showed any cytotoxicity compared with that of TCPS (Figure 7a), as the cell viability in each group was similar. The 90G10C group showed moderate cytotoxicity with less than 30% of the cells not being viable. In general, the degree of crosslinking will increase with increasing exposure time and tyrosine content. However, in the present work, a major dityrosine bond exists between the gelatin molecules, which indicates that the tyrosine content in the 90G10C gel was lower compared to the other gels and that the collagen fibril structure limited the dityrosine bond formation. Therefore, the 90G10C composite contained more unused Ru(II)bpy_3_^3+^, which could be extracted and resulted in lower cell viability compared to the other groups. Nevertheless, the fibroblasts showed a similar morphology in all groups (Figure 7b).

### 3.3. Effect of MBG Particles on the Mechanical Properties of Hydrogel

**Figure 8:**
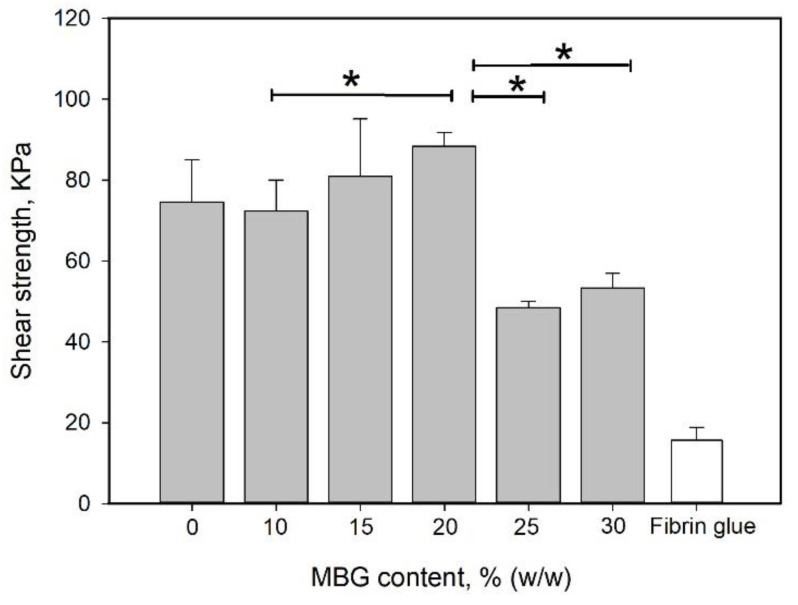
Effect of MBG content on shear strength. MBG was added to and mixed well with the 90G10C solution to a content ranging from 10 to 30% (*w*/*w*). Then, 100 μL of the mixed solution was applied to the porcine skin strips. The 1 cm^2^ overlap area was irradiated by an LED curing light for 120 s. Data are presented as the mean ± SD (n = 9) and were analyzed using the nonparametric Kruskal–Wallis H-test. Differences of *p* < 0.05 were considered statistically significant. (*) denotes a significant difference (*p* < 0.05).

MBG can absorb water and undergo hydrolysis; thus, it is considered a degradable bioglass. Therefore, the water absorption capability of the MBG-containing gel was higher than that of the non-MBG-containing gels (Figure 5). Additionally, MBG did not interfere with the structural integrity of the gel (Figure 2d). Either an increase in the reinforcement material volume fraction or a decrease in the particle size can enhance the strengthening effect of the particulate dispersion [60,61]. The effect of the MBG content on the shear strength is shown in Figure 8. The shear strength increased with increasing MBG content up to 20% (*w*/*w*) and then decreased. The volume ratio of the particle dispersion–strengthened matrix has an optimal value based on the composition of the materials. The particles in the matrix act to prevent crack migration; thus, if the content of particles is too high, the particles become a minor domain rather than a hindrance. This phenomenon can explain why the shear strength decreased with MBG content higher than 20% (*w*/*w*).

### 3.4. Characterization of the Photocrosslinking Hydrogel Reinforced by Nanoparticles and Fibrils

**Figure 9:**
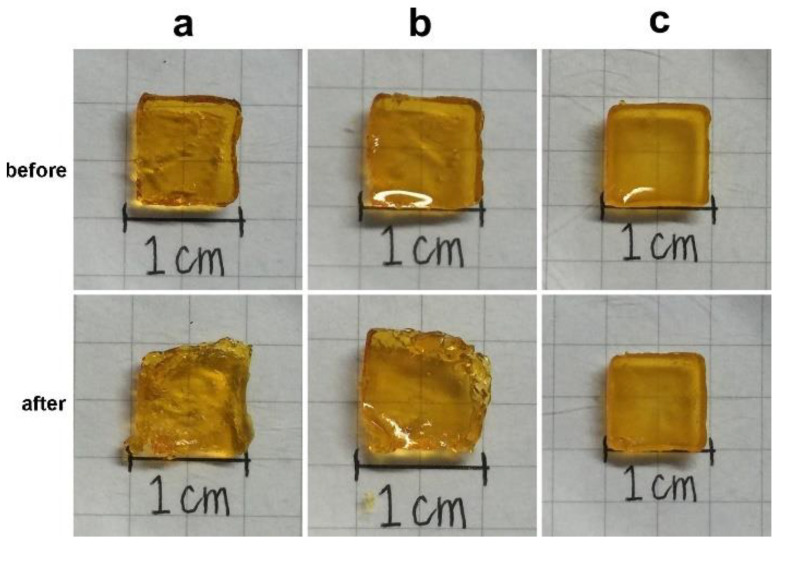
Images of sample used in the compressive test. The gel samples, (**a**) gelatin, (**b**) gelatin containing 20% mesoporous bioactive glass (MBG), and (**c**) 90G10C containing 20% MBG, were irradiated by an LED curing light for 120 s (before) and then were placed on the stage of the material testing system machine (MTS) under compressive test (after).

Figure 9 demonstrates the synergistic effect of collagen and MBG on the mechanical properties of the composite gel. The image was taken when the gel sample was fully compressed, followed by release of the force. As shown in Figure 9b, the gel shape became irregular even for gels containing MBG particles with 120 s of exposure. However, the compression force did not cause significant cracks in the sample containing collagen and MBG. These tensile and compression tests indicated that collagen and MBG enhanced the composite gel stiffness and elasticity. 

**Figure 10:**
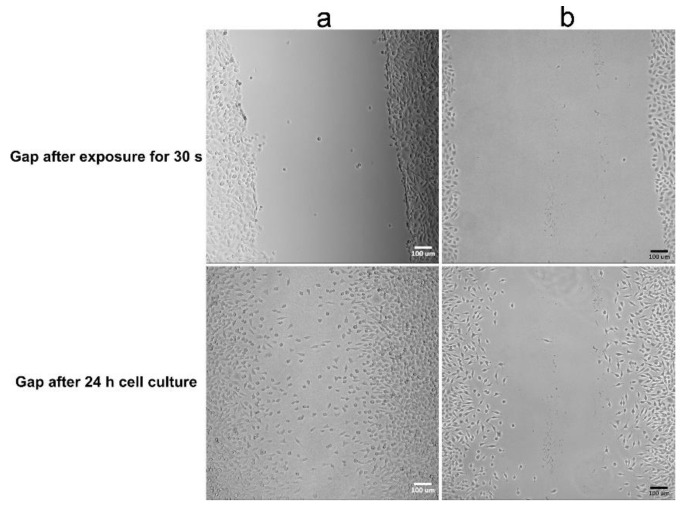
Images of fibroblast cells crossing a gel-covered area. A straight linear gap was created in a confluent L929 cell monolayer. The aqueous composite was placed within the gap area and irradiated for 30 s. Then cell migration was observed with (**a**) 90G10C containing 20% MBG gel, and (**b**) blank used as control.

The components of the gel used in the present work are all biodegradable materials, which means that the sealant will degrade with time alone and will be replaced by tissue. However, in addition to wound healing, preventing adhesion is another important issue. Thus, a gel layer was placed in a gap in an L929 fibroblast monolayer to observe cell migration and to evaluate the gel’s ability to prevent fibroblast invasion. Figure 10 shows that the migration distance of fibroblasts crossing the gel-filled gap was shorter than that of fibroblasts crossing the untreated gap (blank gap). Moreover, the orientation of cells migrating across the blank gap was observed to be directed toward the center, but in the gel group, no consistent cell orientation was observed. The images indicate that a highly hydrated gel can reduce fibroblast migration and alter fibroblast orientation. Cell adhesion and migration are mediated by adhesion receptors linking the cell with ECM ligands and can result in different degrees of extension. Therefore, substrate stiffness and mechanotransduction play an important role in the biological processes of cells, including adhesion, migration, proliferation and differentiation [62]. One possible reason for the finding shown in Figure 9 is that the composite gel has lower compressive modulus levels compared to TCPS, which reduces intracellular force generation and then limits cell migration. Although the cell viability data indicate that the 90G10C composite induced moderate cytotoxicity, Figure 10 shows that the fibroblasts in the 90G10C group still showed the typical morphology with lamellipodal spreading, proliferation and movement outward from the boundary.

## 4. Conclusions

While in situ photocrosslinking methods for hydrogel materials have many advantages, they can also introduce problems, such as tissue damage due to UV exposure. The degree of photocrosslinking depends on the irradiation time and the concentration of photosensitive molecules. Therefore, shortened irradiation time decreases the photocrosslinking degree and then caused poor mechanical strength. In the present study, the sol-gel transformation was activated by visible-light-induced photocrosslinking between native proteins without any molecular modification, and continuous fibrils and particles were used to reinforce the matrix stiffness and hydrostatic resistance to compensate for the short exposure time. The tensile strength of the gelatin/collagen/MBG sealant formed by dityrosine crosslinking was significantly higher than that of fibrin glue. The gelatin/collagen/MBG composite sealant could be used not only to bind two surfaces but also to form a barrier against fibroblast invasion. Together, these results demonstrate that gelatin-based hydrogels reinforced by collagen fibrils and MBG cured in situ by visible light have potential for application as tissue adhesives.

## Supplementary Materials

The following are available online at https://www.mdpi.com/2073-4360/12/5/1113/s1, Figure S1: Effect of irradiation time on gelatin gel weight.
