# Supplementary material for: Gelatin Hydrogels Reinforced by Absorbable Nanoparticles and Fibrils Cured In Situ by Visible Light for Tissue Adhesive Applications
Source: Polymers (Basel). 2020 May 13;12(5):1113. doi: 10.3390/polym12051113 (PMC7285276; doi:10.3390/polym12051113)
Supplement: Supplementary file 1 [file polymers-12-01113-s001.pdf]

Supplementary Materials

The gelatin gel samples, which were irradiated for a designated time, were immersed in an artificial saliva solution (pH 7), and weighted every 24 h.

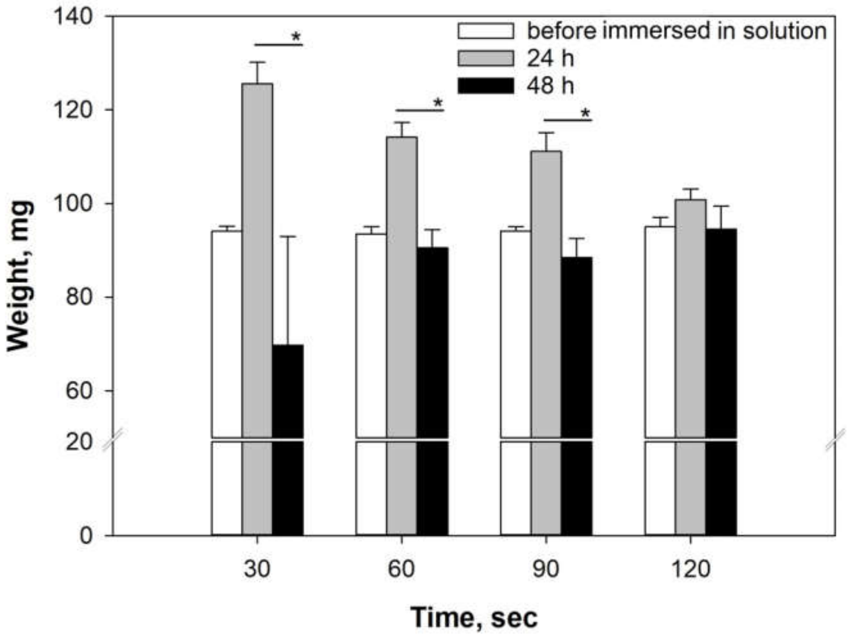

Figure S1: Effect of irradiation time on gelatin gel weight. Data are presented as the mean ± SD (n=9) and were analyzed using the nonparametric Kruskal–Wallis H-test. Differences of $p<0.05$ were considered statistically significant. (*) denotes a significant difference ($p < 0.05$)
